# Unexpected bat community changes along an urban–rural gradient in the Berlin–Brandenburg metropolitan area

**DOI:** 10.1038/s41598-024-61317-7

**Published:** 2024-05-08

**Authors:** Nicole Starik, Lorenz Gygax, Thomas Göttert

**Affiliations:** 1 Albrecht Daniel Thaer-Institute of Agricultural and Horticultural Sciences, Faculty of Life Sciences, Humboldt-Universität zu Berlin, 10009 Berlin, Germany; 2 Deutsche Fledermauswarte e.V., Am Juliusturm 63, 13599 Berlin, Germany; 3 Research Center [Sustainability-Transformation-Transfer], Eberswalde University for Sustainable Development, 16225 Eberswalde, Germany

**Keywords:** Urbanization gradient, Urban winners and losers, Anthropogenic noise, Artificial light, Bioindicators, Urban ecology, Animal behaviour

## Abstract

Urbanization gradients are increasingly used in ecological studies to discover responses of species communities to different intensities of human-induced habitat transformation. Here, we investigated patterns of bat communities against the background of different urbanization levels using a priori defined urbanization categories based on distance classes (5 km intervals) along a linear transect from the urban core of the city of Berlin westwards into the rural outskirts of the state of Brandenburg. Using linear-mixed effects models, we found that “distance class”, as a proxy for urbanization level, is a meaningful and suitable predictor of bat species richness and diversity. We observed an unexpectedly sudden increase in bat species richness and diversity and changes in species-specific activity levels relatively close to the urban center at the transition between urban and peri-urban areas. This change suggests a relevant influence of the peri-urban areas as a “buffer zone” for specific bat species not able to adapt to the heavily modified inner core of the metropolitan area. Although we could demonstrate that anthropogenic noise and artificial light have the potential to predict the variability of bat species activity along the urban–rural gradient, the actual influence on observed shifts in the bat community needs further research.

## Introduction

Rapidly expanding urban landscapes represent novel and challenging ecosystems for wildlife, and in some cases, they can even foster the rise of wildlife-directed conflicts (human-wildlife conflicts sensu Göttert and Starik)^1^. These human-constructed landscapes do not only contain artificial infrastructures, such as buildings and roads, but are characterized by completely new environmental conditions^2^, such as increased anthropogenic noise and artificial light levels^3–5^. On a global scale, effects of urbanization on biodiversity are detrimental, leading to increasing functional, taxonomic and genetic similarity of areas over time (“biotic homogenization”). One reason for this homogenization is that species richness tends to decrease towards more urbanized areas for most systematic groups^6,7^. This has been illustrated by several studies using the example of vertebrates, including songbirds^8,9^ and bats^10^. Following Czech et al. urbanization can therefore be seen as “*a major cause of native species extinction*”^11^.

With regard to bats, the majority of studies suggest a negative association between overall bat species diversity and urbanization level, which seems consistent in cities with different characteristics and ecological contexts^12–15^. At a local scale, however, responses to urbanization intensity may vary among species. Moreover, some bat species appear to even thrive in urban environments by adjusting their behavior and ecology according to the changing environmental conditions^5,16^. Despite the generally adverse conditions in urban environments, new opportunities may arise for so-called tolerant species^17–19^. This seems to apply also to bat communities on a global scale^10,12,19,20^: a few “urban winners” can be found in high abundances, while the more sensitive species, the “urban losers”, do not manage to persist in the city and refrain from urban areas. Following this, it is not only the decrease in species richness that contributes to the phenomenon of “biotic homogenization”, but also the non-random distribution of "winners" and "losers", the few winners being those species that thrive in environments disturbed by humans^21^.

Previous research highlights different factors that might contribute to the negative effects of urbanization on bat species. For instance, while for some species, prey seems easily accessible in cities due to the large number of insects attracted by streetlamps^19,22^, artificial light at night (ALAN) may negatively affect foraging behavior, roosting, and hibernation of other species^4,23–25^. Also, obligate tree roosting bats might not find enough opportunities in urban environments. However, at the same time, man-made structures, such as buildings, roofs and tunnels (artificial roosts) with their warmer microclimatic conditions, may benefit species which are not necessarily dependent upon tree roosts^19^. In addition, urban anthropogenic noise may affect the foraging success for some species, as noise frequencies often overlap with the frequencies of sounds emitted by bat prey^3,26–28^. Similarly, noise might affect communication with conspecifics by masking some bat species’ social calls^26^. Other factors that can affect bats in urban environments include roads^29^, predation by domestic animals^30^, or the urban heat^31^. Thus, on the one hand, ecological differences among species and the degree of a species’ ecological flexibility (plasticity) result in a different sensitivity towards human-induced disturbances. On the other hand, the presence and distribution of wildlife, including bat species, is largely affected by the intensity of anthropogenic land use (urbanization level)^32–35^.

To better understand the relation between environmental variation and the structure (and function) of communities, ecologists have effectively studied natural gradients in the past^36^. Approaches to gradually investigate urban to rural differences in bat communities seem promising to reveal knowledge on the adaptability of different bat species to urbanization. Due to the nature of most cities, a classical gradient of land use intensity and biodiversity can rarely be found. While most cities are characterized by a highly transformed urban core, this center is rarely surrounded by symmetric rings of diminishing landscape modification. If this is nevertheless the case, the resulting array of natural and human-modified ecosystems within a metropolitan area can be conceived of as a readily measurable gradient of land use and a more complex gradient of urban effects^37–39^. In these cases, the gradient paradigm seems a useful tool for research on the ecological consequences of urbanization. For example, Berlin, Germany's largest city, offers particularly suitable conditions for studying the classic interplay between urbanization intensity and biodiversity due to its historical growth and spatial structure (metropolis surrounded by a low-population matrix).

Within the current study, we investigated the spatial patterns of bat communities in urban–rural landscapes against the background of the different levels of urbanization in the city of Berlin and the surrounding federal state of Brandenburg. Berlin is a metropolitan city and the surrounding areas are characterized by agriculture and a low density of human settlement. Since all European bat species are legally protected as of Annex IV of the Habitats Directive of the European Union (Council Directive 92/43/EEC), these results are essential to develop species-specific conservation strategies and to mitigate the effects of anthropogenic disturbances on bats in urban areas. We suspected that the structure of the Berlin–Brandenburg bat community changes from urban to rural habitats. Therefore, the overall aim of our study was to describe if and where this potential change at the urban–rural interface occurs by using a comprehensive spatial scale.

To identify possible changes in bat community parameters, we measured the variation in species diversity and richness and the species-specific activity across an urban-to-rural gradient using a priori defined urbanization categories along a simple, linear transect from the urban core of the city Berlin westwards into the rural outskirts of Brandenburg. Given the prevailing negative association between overall bat species richness and urbanization level^12–15^ and the fact that some species are more sensitive than others to urbanization^19,40^, we predicted that likewise, bat species richness and diversity increase with increasing distance from the city center. In other words, we predicted a gradual turnover in bat community characteristics along the gradient. By doing so, we aim at providing additional evidence of the detrimental impact of urbanization on bats, thereby contributing to the existing body of literature on the subject, which needs to be consolidated as shown by Browning et al.^41^.

Although it is very difficult to estimate the importance of different anthropogenic disturbance factors that impair the different bat species’ survival in urban environments, particularly because several of them can operate simultaneously, specific anthropogenic disturbance variables (i.e. noise and light conditions) are known to have a measurable negative impact on bat activity (e.g.^3,4,24–27^). Thus, it can be assumed that such continuously measurable disturbance variables might reflect and define the degree of urbanization more accurately compared to simple urbanization categories. Therefore, we finally aimed to investigate whether community parameters (reflected by species richness, species diversity and species-specific activity) could be predicted more precisely by one of the anthropogenically-driven variables ambient noise and artificial light compared to urbanization categories based on a linear geographical gradient.

## Materials and methods

### Study area and sampling sites

**Figure 1:**
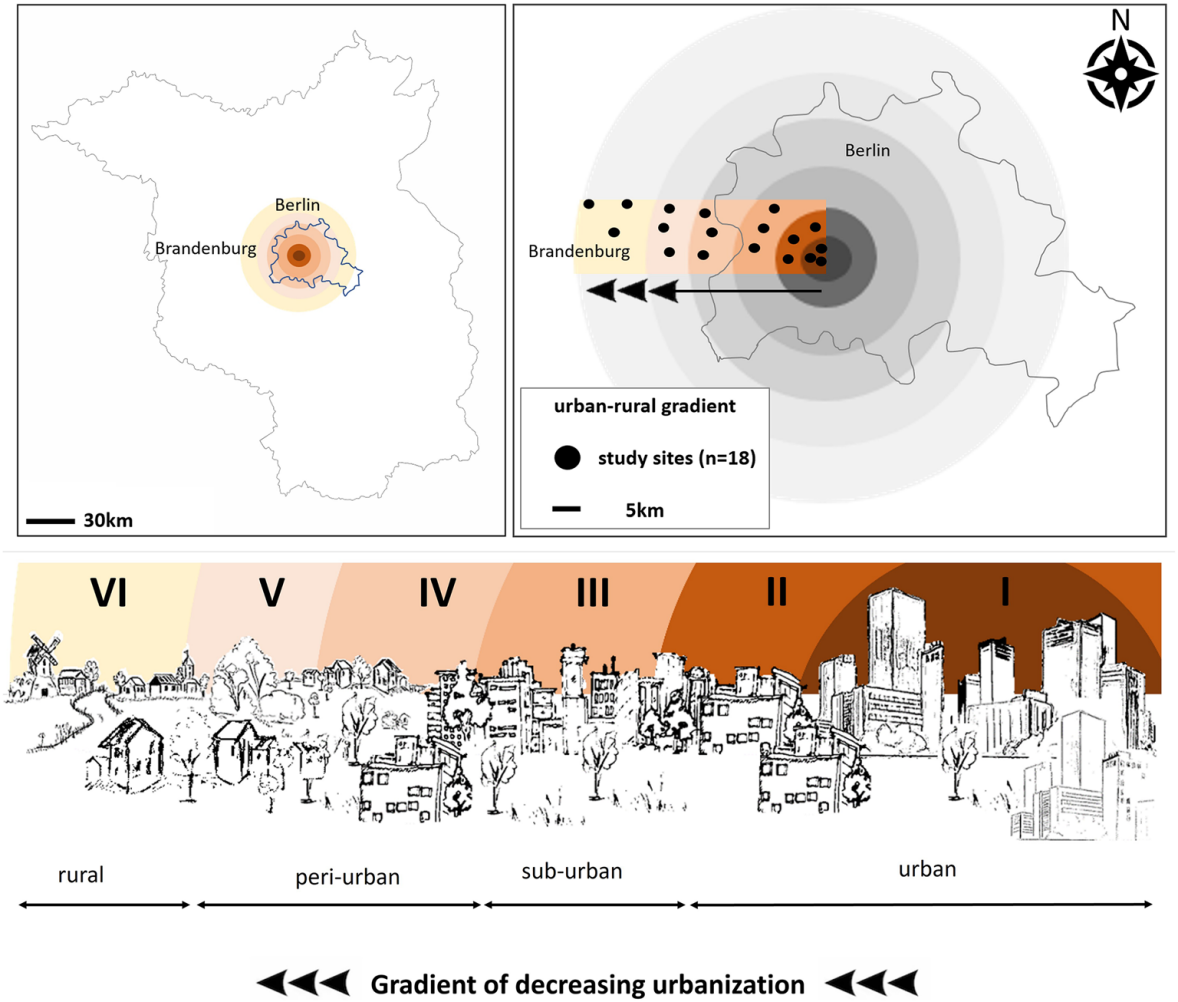
Location of the study sites along a linear urban–rural gradient from Berlin city into Brandenburg. Along this gradient, six distance classes at 5 km intervals were defined to reflect the decreasing urbanization level. Within each distance class, we established 3 sampling sites, which were monitored once per week between April and October 2020 and 2021 and were considered to be independent. Arrows indicate the westwards directed urban–rural gradient and define x-axes in subsequent figures, (I–VI). Distance classes I–II: urban; distance class III: suburban; distance class IV–V: peri-urban; distance class VI: rural. The external borders of the district of Berlin and the federal state of Brandenburg were created on the basis of this file: https://en.wikipedia.org/wiki/States_of_Germany#/media/File:Germany_location_map.svg (NordNordWest, CC BY-SA 3.0); the rest of the figure was hand drawn by one of the authors (N.S.).

The study area was situated along a transect of increasing distance from the city center of Berlin capital city (Germany; 52°523′ N, 13°397′ E) westward into the rural surrounding of the federal state of Brandenburg (52°497′ N, 12°925′ E) (Fig. 1). A total of 18 green area sampling sites (all of them were parks) were chosen stratified within six distance classes (distance classes I-VI) along this gradient at relatively small distances to each other such that the study remained practically feasible. This resulted in three sampling sites (replicates) per distance class, separated by at least 500 m. Given that (1) the differences in distances to the town center are very small between the sites of a given distance class in comparison to the sites of different distance classes (as seen in Fig. 1) and that (2) the distances to the town center were very similar between sites in a distance class, the distance classes contain virtually the same information as the real (continuous) distances.

### Site characterization and anthropogenic disturbance factors

In order to identify environmental conditions, which are likely to affect the access to important resources for bats and to characterize the level of urbanization and/or disturbance, several variables were assessed (Table S1). Distances to nearest water source, trees, and buildings were calculated in QGIS software v. 2.18.0 (QGIS Development Team, 2018). Proportion of impervious land cover surrounding each site (landscape scale) was measured within a 500 m radius with publicly available data from the Berlin Environmental Atlas^42^ at a 2 × 2-m resolution and using the Zonal statistics tool in QGIS. We chose a 500 m radius buffer because it encompasses the general foraging range of most bat species identified and is likely to serve as a medium spatial scale for assessing bat-habitat relationships^43^. For all sites, the height of all trees and buildings within a 100 m radius were measured using a laser range finder (LRF 600, Walther GmbH, Germany) and averaged to a mean value. During five full nights within the study period, both ambient noise (as a proxy for anthropogenic noise pollution) and the brightness of the night sky (as a proxy for anthropogenic light pollution^44^) were measured on each site. Measurements for noise and sky brightness were taken once every full hour starting 60 min before sunset until 60 min after sunrise, and averaged to a mean value per night. We did not collect data on all 18 sites at a time due to logistic constraints. Anthropogenic noise was measured using a commercial sound level meter (SLM, Model: Lutron, SL-4001), which generated calibrated measurements of the ambient sound pressure level (SPL; dB re 20 μPa). We describe the level of light pollution by measuring the sky brightness using a highly sensitive sensor (Sky Quality Meter SQM, Unihedron, Canada) during nights of clear sky (not overcast). This sensor acquires measurements of the zenithal sky brightness at a solid angle of 42° around the central axis (full width at half maximum) resulting in magnitudes per arc second^2^ as values. Values of the sky quality meter are inversely proportional to light; hence, higher digits indicate less light intensity. Whenever levels of light and noise were assessed, acoustic monitoring was also carried out simultaneously (see “Bat surveys”). To ensure that all explanatory variables are recorded at the same time, all observations were synchronized. 

### Bat surveys

For an autonomous continuous monitoring of bat species throughout entire nights^45^, we deployed automatic passive acoustic recorders with omnidirectional ultrasonic microphones (batcorder types 2.0 and 3.0; ecoObs GmbH, Nuremberg, Germany) once a week at each of the 18 sampling sites. The measurements were conducted in two subsequent years between April to October 2020 and 2021. Within each site, we placed one batcorder 2.5 m above ground on a pole. The recorders were scheduled to record from 60 min before sunset until 60 min after sunrise following standard protocols for acoustic bat surveys (e.g.^46^). A total of 54 weekly surveys (repeated measurements; 27 weeks in both years) were conducted per site. Six sites (with random selection of one site in each distance class) were sampled in parallel during every recording night so that all locations were sampled for the same number of nights. We sampled data during adequate weather conditions (no rain and wind speed < 6 m/s predicted in the weather forecast^47^) and deployed devices oriented away from clutter (no foliage, branches, buildings, etc.) to avoid echoes. We did not consider the lunar cycle within our study design, since previous studies on bat “lunar phobia” provide contradictory evidence suggesting divergent geographical and species-specific patterns^48^: while for some species moonlight intensity influences activity (e.g.^49,50^), this effect could not be confirmed for other species^51–53^. We made full spectrum recordings in .raw format [sampling rate: 500 kHz, record quality: 20, amplitude resolution: 16 bit, threshold amplitude (sensitivity): − 36 dB, post trigger: 400 ms, threshold frequency (sensitivity): 16 kHz]. Each sampling location within a given sampling site was selected such that they all had a similar maximum distance to roads (45.8 ± 17.7 m) and street lighting (55.6 ± 16.8 m).

### Acoustic data analysis

A total of 72,589 recordings of bat calls were analyzed using the software bcAdmin (Version 3.6; ecoObs GmbH, Nuremberg, Germany) followed by automated species identification with the software bcAnalyze (Version 3 Pro; ecoObs GmbH, Nuremberg, Germany) in combination with the software batIdent (Version 1.5; ecoObs GmbH, Nuremberg, Germany). This approach is based on Random Forest classifications (in R^54^) and results in assignment probabilities for the calls of a sequence for up to three species per recording. Bat calls that cannot be assigned to species level due to a similar call structure and/or frequency range are grouped (i.e., pooled) to sonotypes. Depending on the level of accuracy, the internal classification algorithm of batIdent does so for the sonotypes “Plecotus” (containing *Plecotus auritus* and *Plecotus austriacus*), “Nyctaloid” (containing *Eptesicus serotinus*, *Vespertilio murinus*, *Nyctalus leisleri*, and *Nyctalus noctula*), “Myotis” (containing all *Myotis* species) and “Pipistrelloid” (containing all *Pipistrellus* species). Given sufficient assignment probability, the algorithm may also determine up to the sonotype levels “Nycmi” (containing *N*. *leisleri*, *E*. *serotinus* and *V*. *murinus*), “Nyctief” (corresponds more or less to *N*. *noctula* in our study area) and “Mkm” (containing *Myotis mystacinus*, *Myotis brandtii* and, in some cases, *Myotis daubentonii*) or even to sonotype levels “Pmid” (corresponds more or less to *Pipistrellus nathusii* in our study area) and “Mbart” (*M*. *mystacinus* and *M*. *brandtii*)^55^. Because there is evidence that automatic species identification is subject to a high rate of misidentification^56–58^, we manually validated all the softwares’ automatic output with classification probabilities below 95%. Using the software BatSound ver. 4.1.4 (Pettersson Elektronik AB, Uppsala, Sweden), we post-validated these low classification probabilities (n = 26,858) and all sonotype level classifications comparing measured parameters on spectrograms (shape, peak frequency, duration, intervals) on the basis of the most distinctive call types with those available from the literature^59–62^. However, overlap in call characteristics still made it difficult and sometimes impossible to distinguish closely related species by their echolocation calls^61^. Therefore, calls, which still could not be identified to species level by manual inspection were left grouped respective to sonotypes. For all automated detections at the level “Nyctaloid” we were able to attribute the calls to species level (Nnoc, Nlei, or Eser). For almost 50% of the automated detections on the level “Nycmi” we were also able to manually identify the species. Due to the high similarity in call structure among species, the sonotype “Plecotus” and “Mkm” can hardly be separated acoustically and were used accordingly in the analysis on this lowest possible identification level.

As a simple metric of bat species diversity, we calculated species richness (number of species verified through manual classification, excl. sonotypes “Plecotus”, “Nycmi” and “Mkm”) per sampling site for each sampling night. In order to describe alpha diversity of the bat community for each sample night and to provide a measure of the evenness of the community, we furthermore calculated the Shannon index (referred as "species diversity" in the rest of the manuscript). This index takes into account both the number of species and their relative abundances [H = − Ʃpi × ln (pi), where pi represents the proportion of total activity represented by species “i”]. For this calculation, we defined the total bat activity as the number of 1-min intervals with at least three identifiable bat calls of a given species (also excl. sonotypes) at a given sampling site (= “active minutes”). Total bat activity thus contained search-phase, foraging and social calls.

In an attempt to estimate the approximate dimensions of “foraging activity” of bats along the gradient and following Brigham^63^, we reviewed all manually classified recordings (incl. sonotypes) for the presence of feeding buzzes. Feeding buzzes have a characteristic increase in pulse rate and drop in terminal frequency compared to search-phase calls and indicate foraging behavior at a site, either actual prey capture or attempted capture events^64^. Thus, for our purposes, foraging activity was defined as the number of 1-min intervals during which a feeding buzz of a given species at a given sampling site occurred (whenever feeding buzzes were observed in a call sequence, we counted this sequence as “foraging”). In contrast, the number of 1-min intervals, during which a search-phase call of a given species at a given sampling site occurred without indication of subsequent feeding buzzes was defined as “commuting activity”. As there is strong difficulty in assigning a given call sequence to actual behavioral categories on the basis of feeding buzzes alone, the results and conclusions should be interpreted with care. It should be noted that in some cases, a sequence without feeding buzzes must not necessarily represent commuting behavior only because some species do not emit the typical feeding buzz due to other foraging strategy and/or echolocation behavior^65^. Also, feeding buzzes may be used by some bats when drinking and landing and thus must not necessarily represent foraging activity only. In order to make nights of different lengths comparable, we used an activity index^66^, which was calculated by dividing the number of active minutes (with either commuting or foraging calls) by the number of minutes of monitoring (sampling effort)^66,67^. The final index thus described the proportion of minutes with a specific activity for each species, site and night.

### Statistical methods

All statistical evaluations were conducted in R Version 4.3.1 (R Core Team, 2023). We used principal component analyses from package stats (part of the R base installation) and generalized mixed effects models from package blme (^68^; based on package lme4^69^).

### Collinearity of predictor variables and choice of fixed effect(s)

First, we investigated the collinearity of potential predictor variables that described the urbanization gradient. Two sets of predictor variables were available in our data set. The first included variables specific to each site. These variables did not change throughout the investigation (see site-specific characteristics, Table S1). The second set included variables that varied from night to night (night-specific characteristics, i.e., ambient noise and sky brightness). Each of these two sets of variables was complemented by a variable reflecting the urbanization gradient from 1 (urban core: ≤ 5 km distance to geographical city center ≙ distance class I) to 6 (most rural: ≥ 30 km distance to geographical city center ≙ distance class VI). As argued above, the distance classes reflected the true distances to the city center closely. Accordingly, we used the distance classes as a numerical continuous variable here with the values 1–6 (values that were proportional to the true distances to the city center). For each set of potential predictor variables, a principal component analysis (PCA) was conducted.

For the first dataset, the first two principal components (PCs) represented 53 and 17% of the total variance (70% in sum). The first PC was positively associated with distance class, and negatively associated with impervious surface and mean height of buildings (Fig. S1, Table S2). The second PCA component increased with the values distance to trees, distance to buildings and distance to water. Accordingly, the two components might have captured independent characteristics of the environment. Given that the second PC only accounted for 17% of the total variance and only a third of the first component, it was likely to have a smaller influence on the outcome variables further investigated (see next section). Moreover, all these characteristics are site-specific resulting in a sample of only 18 observations heavily limiting the number of potential predictor variables that could be investigated at site-level. Accordingly, the current sampling scheme was not designed to evaluate differences due to site specific variables. This is why we chose to investigate distance class as a representative variable of the first PC. We consider the second PC (and the additional variables) as aspects that characterize the sites and that, accordingly, set the frame to what kind of sites the results of this study could be extrapolated.

Using the second data set, the first component represented 93% of the total variance and was positively associated with distance class and with sky brightness (inverse measure of light pollution), and negatively associated with ambient noise (see Table S2). Based on this analysis, we concluded that the urbanization gradient based on distance classes could well represent all three variables. Because this gradient was available for all our observations, we used it as the main predictor variable in our models on the effects of urbanization on bats (see next section). In addition, we investigated how well sky brightness or ambient noise could replace the distance class as a predictor variable in the reduced data-set (see below).

### Effects of different urbanization levels on bats

Distance class was used as the predictor variable in two models with a similar structure. In this part, we used the variable distance class as a categorical variable (with six levels I–VI). This was mainly due to the fact that this approach of coding distance class allowed for a more flexible and potentially non-linear shape in the relationship of the distance class with the outcome variables. Accordingly, the influence of the urban–rural gradient can be estimated more exactly. We considered the number of species observed in a given night as the number of “successes” and the total number of species/sonotypes observed during the study (N = 12) as the number of “trials”. These successes and trials representing species richness were used as the outcome variable in a model based on the binomial distribution and the logit link-function. Also, the Shannon diversity index was used as the outcome variable in a linear mixed model with normal errors. In both models, site identity and the calendar date were both included as crossed random effects and coded as categorical variables.

To further investigate species-specific effects, the activity index was used as the outcome variable in an additional model with normal errors. To satisfy model assumptions, this proportion was logit transformed. To be able to use this transformation, we had to replace the 0 values that were observed. For each species, we defined the detection threshold as the smallest activity index observed that was larger than 0. We then replaced the zeros by a value 10% smaller than the detection threshold. If at all, this makes the data set slightly less extreme and can be considered accordingly a conservative approach. In this model with the activity index as the outcome, the distance class (categorical variable with six levels), the species/sonotype (categorical variable with twelve levels), the context (categorical variable with two levels: commuting and foraging), and all their interactions served as predictor variables. The site identity and the calendar data were again used as crossed random effects and were coded as categorical variables.

We checked residuals graphically for normality and homoscedasticity using the package DHARMa^70^. In addition, we checked random effects for normality using quantile–quantile plots. Except for the model of the Shannon diversity index, we found some indication of a narrow error distribution with long tails possibly due to the observations without certain bat species, i.e. an activity of zero. Because the model estimates followed the raw data very closely (as visualized in our results figures), we considered the deviations as minor and without the need to adjust the models.

P-values were calculated using a parametric bootstrap approach as provided in the package pbkrtest^71^. To be able to interpret the p-values of main effects even in the presence of interactions, we used sum contrasts for all our categorical predictor variables and normalized values for all our continuous predictor variables. Model estimates and confidence intervals were also based on a bootstrap approach using some additional facilities from the package boot^72,73^.

### Choosing the optimal predictor variable

For some nights (see above), night-specific measurements of noise and light pollution were available. In an attempt to investigate the respective importance of the effects of light, noise, and other characteristics that may be reflected by the distance class, we calculated three additional models. Apart from the smaller number of available nights, the first model was identical to the ones described for species richness, the Shannon diversity index, and the activity index above. In the second and third model, the distance class was replaced either by the light or by the noise measurements, respectively. Both these variables were used as continuous predictor variables.

Whereas the linear component for the light and noise measurements was sufficient on the (transformed) scale of the model of species richness (due to the use of a logit link function), the model of the Shannon diversity index needed to include a cubic and a quartic polynomial function for noise and light, respectively, to allow for capturing the non-linearity of the relationship. In other words, these polynomials were necessary to no longer see any non-linear structure in the plots of the residuals versus noise and light intensities. Moreover, the random effect of calendar date was dropped in the models of species richness and the Shannon diversity index because only one observation had taken place per night on this level of data aggregation. We included also additional quadratic terms for light or noise in the two models with the activity index as the outcome variable due to non-linearity of the relationship.

We then compared the three models (distance class, noise, light) based on the Bayes information criterion (BIC). Given the models described above, the full models for species richness used 7, 3, and 3 degrees of freedom for the fixed effects of distance class, noise, and light, respectively. For the Shannon diversity index, these degrees of freedom amounted to 8, 6, and 7. In the models of the activity index, 147 degrees of freedom were used for the fixed effect of distance class and 75 degrees of freedom each in the model using noise and light intensities as the fixed effects. Due to the number of estimated parameters (and their penalizing in the BIC), the models with distance class were at a a-priori disadvantage. This disadvantage was slight for the models with species richness and diversity and more pronounced for the activity index.

### Ethics statement

Ethical review and approval were not required for the animal study because we passively collected data using only sound recordings and as we did not conduct any collection or handling of animals, our research was not subject to institutional or governmental regulations. Field studies did not disturb endangered or protected species. No privately owned or protected land was accessed during the recordings.

## Results

### Effects of urbanization on bat community parameters

In total, nine species were identified: *Pipistrellus pipistrellus*, *Pipistrellus nathusii*, *Pipistrellus pygmaeus*, *Eptesicus serotinus*, *Nyctalus noctula*, *Nyctalus leisleri*, *Vespertilio murinus*, *Myotis nattereri*, *Myotis daubentonii*). In addition, recordings enabled the detection of three sonotypes: Nycmi (containing *Nyctalus leisleri*, *Eptesicus serotinus*, *Vespertilio murinus*), Mkm (containing *Myotis brandtii*, *Myotis mystacinus*), and Plecotus (containing *Plecotus auritus*, *Plecotus austriacus*).

**Figure 2:**
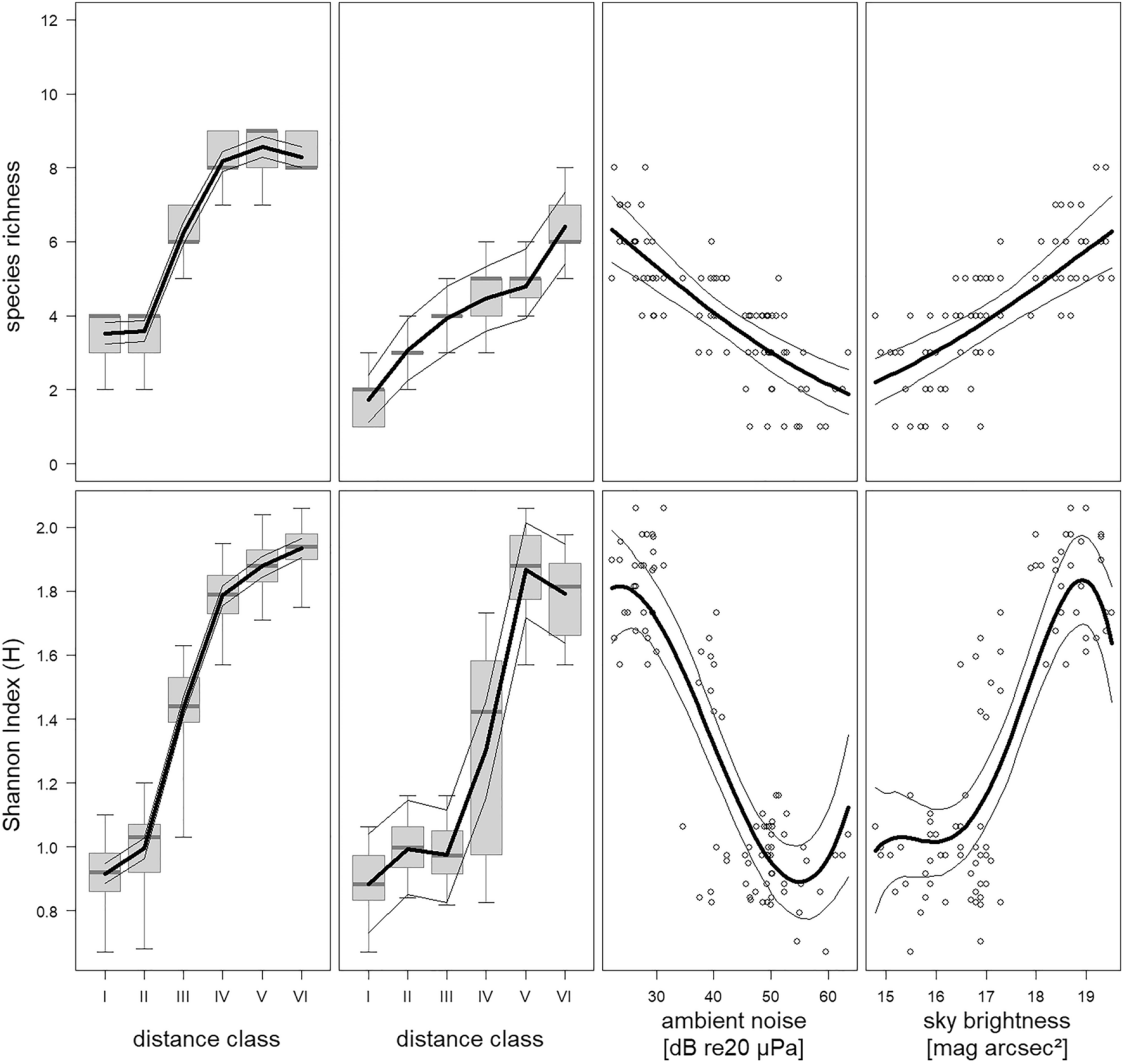
Species richness (number of species observed per night; upper row) and species diversity (Shannon’s H index per night; lower row) in relation to the distance classes (data set 1 with all nights in first left column, data set 2 with the reduced set of nights, during which ambient noise and sky brightness was measured, in second column), ambient noise (data set 2 in third column) and sky brightness (data set 2 in fourth column) reflecting the urban–rural gradient. Boxplots represent medians, quartiles and the total ranges. Thick lines: Model estimate; thin lines: 95% confidence intervals.

Species richness and species diversity increased with decreasing urbanization (main effect distance class p < 0.001 for both outcome variables; Fig. 2, left column). Species richness was low, intermediate, and high for distance classes I–II, III, and IV–VI, respectively. The pattern was similar for species diversity but with some additional differentiation within distances classes I–II and IV–VI.

### Effects of urbanization on species-specific activity

Activity depended on the distance class as well as the species and the context (three-way interaction: *p* < 0.001). In general, commuting was higher than foraging activity (main effect context: *p* = 0.003, Table S3).

**Figure 3:**
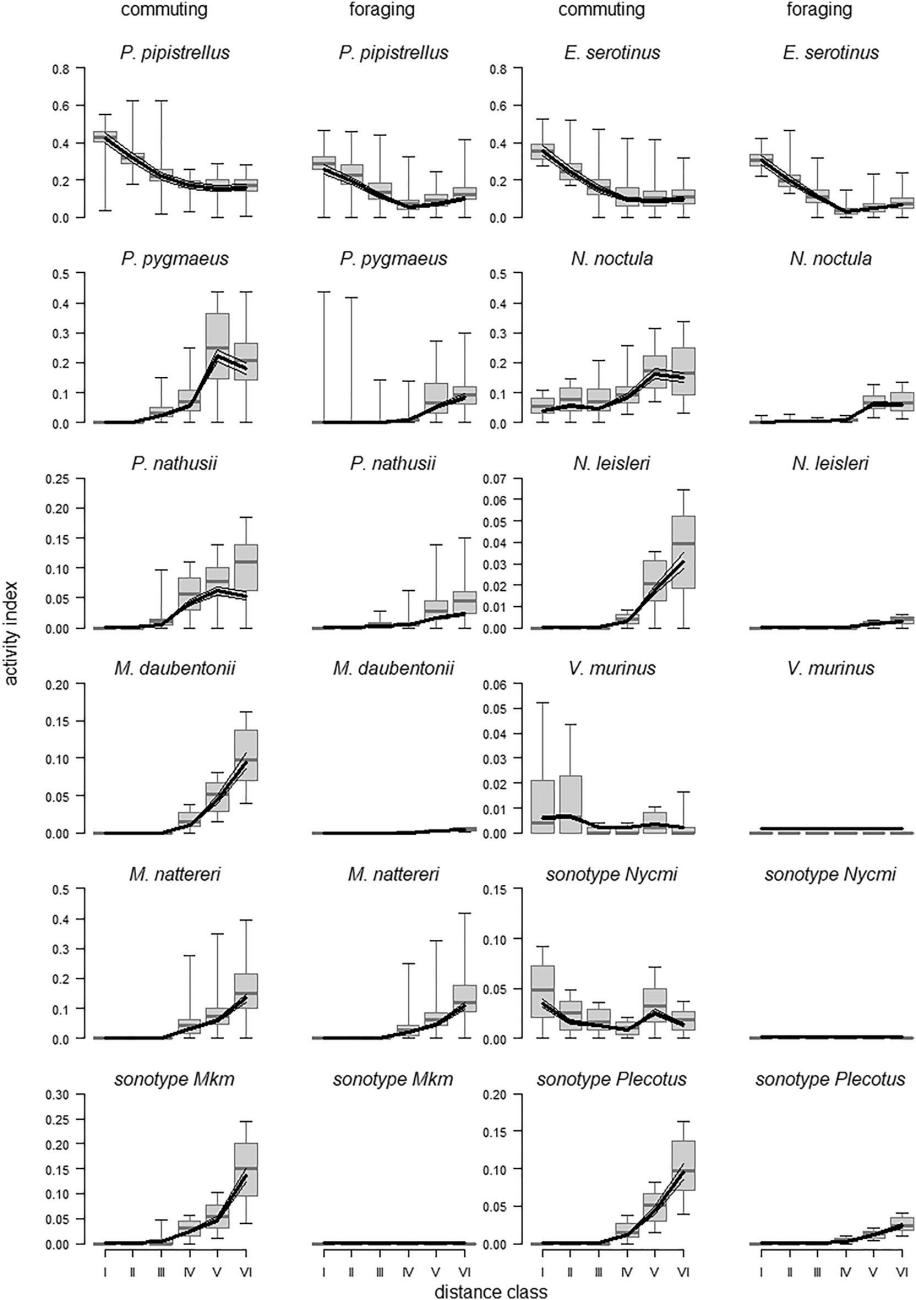
Species-specific commuting and foraging activity of bats along the urban–rural gradient from distance class I (most urban) to distance class VI (most rural). Please note the different scales of the Y-axes. Boxplots represent medians, quartiles and the total ranges. Thick lines: Model estimate; thin lines: 95% confidence intervals.

For 8 out of 12 species/sonotypes activity levels increased with decreasing urbanization (Fig. 3). For *Myotis nattereri* and the sonotype Plecotus, this gradual increase was observed similarly in both commuting and foraging activity. In *Pipistrellus nathusii*, *Nyctalus noctula*, and *Pipistrellus pygmaeus*, activity increased also towards the rural areas. Yet, in these species the increase levelled off at distance classes V–VI. This was specifically visible in the commuting activity. Activity levels of *Myotis daubentonii*, *Nyctalus leisleri* and the sonotype Mkm were also characterized by a gradual increase towards the rural areas, but foraging activity was virtually absent.

Commuting activity in *Vespertilio murinus* and the sonotype Nycmi was low but fairly constant across the urbanization levels but, again, hardly any foraging could be observed. Finally, *Pipistrellus pipistrellus* and *Eptesicus serotinus* were characterized by an increase in both commuting and foraging activity towards urban areas.

### Assessing the influence of the anthropogenically-driven variables ambient noise and artificial light

**Figure 4:**
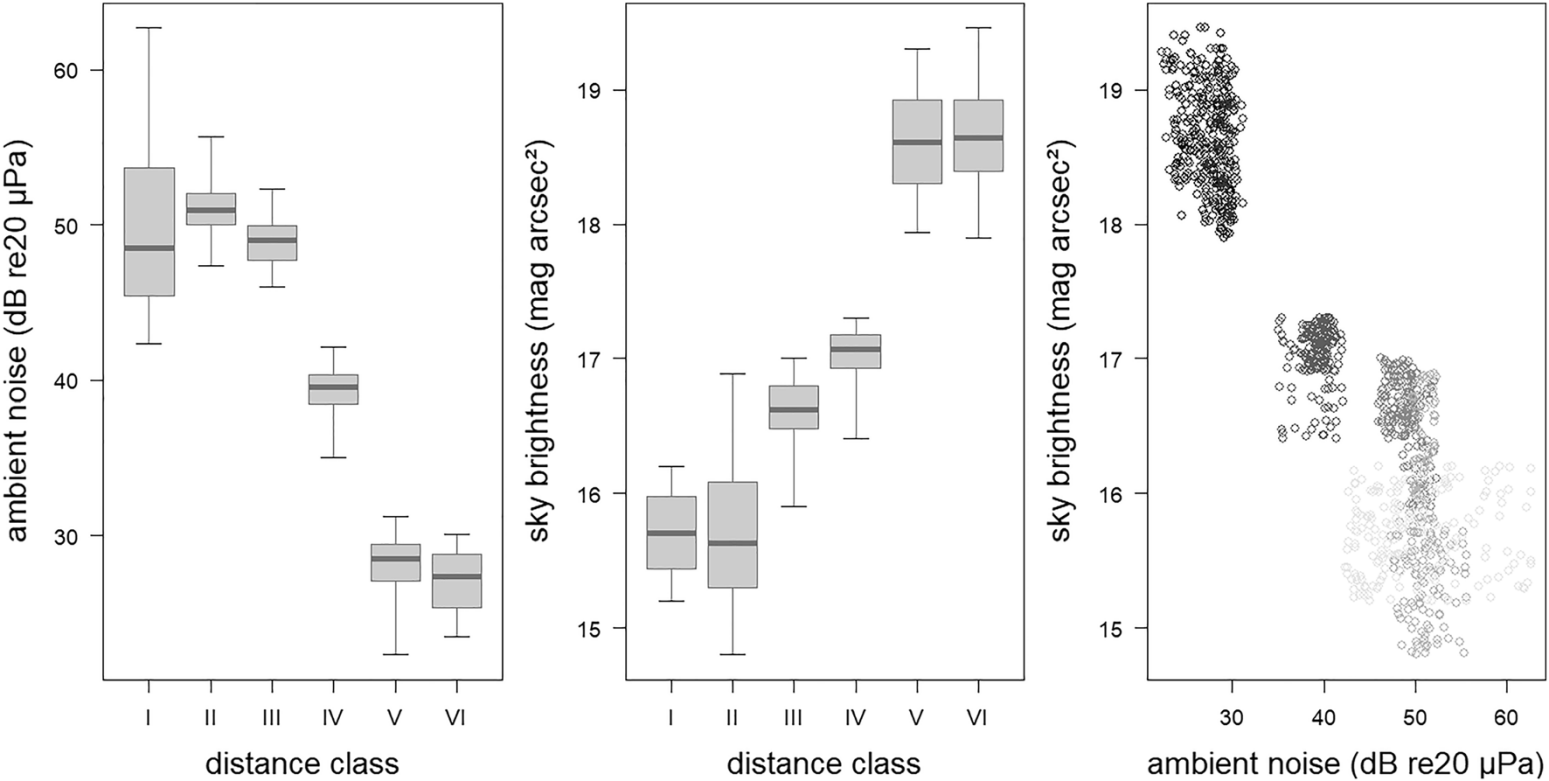
Night-specific observations of ambient noise (left), sky brightness (middle) and scatter plot of ambient noise and sky brightness (right; the darker the dots the higher the distance class) as measures of anthropogenic disturbance for the six distance classes (I–VI) along the urban rural gradient. Boxplots represent medians, quartiles and the data ranges.

Night-specific conditions of the variables “ambient noise” and “sky brightness” as proxies for anthropogenically driven noise and light pollution were investigated along the urban to rural gradient. Overall, these three variables were correlated (Fig. 4, Table S2, Fig. S1b). For both pollution variables, we noted that distance classes V and VI clearly differed from distance classes I–IV (Fig. 4). This distinction became even more pronounced when considering the relationship of these two variables (Fig. 4).

We used these two night-specific characteristics, ambient noise and sky brightness, as alternative explanatory variables to the distance classes when predicting species richness, the species diversity, and species-specific bat activity (Fig. 2, second to fourth column; Figs. S2, S3, and S4) to investigate whether these outcome variables could be predicted more precisely by the anthropogenically-driven variables ambient noise and sky brightness compared to urbanization categories based on a linear geographical gradient.

The model predictions in this reduced data set with distance class as the predictor showed the same qualitative patterns as described above, although the patterns for species richness and the species diversity were not as clear due to the reduction of the data set (Fig. 2, second column).

For species richness, the model using distance class as the predictor fitted the data clearly better (BIC = − 17.5; main effect distance class: p = 0.001) than both models either using ambient noise (BIC = 298.8; linear effect ambient noise: p = 0.001) or sky brightness (BIC = 304.5; linear effect sky brightness: p = 0.002). This was in spite of the fact that even the models using ambient noise and sky brightness seemed to fit the data quite well (Fig. 3, upper row, third and fourth column). Ambient noise could predict species richness slightly better than sky brightness.

For the species diversity, the model using ambient noise (BIC = − 13.4; linear effect: p = 0.001, quadratic effect: p = 0.12, cubic effect: p = 0.001) fitted the data slightly better than the model with sky brightness (BIC = 6.9; linear effect: p = 0.003, quadratic effect: p = 0.005, cubic effect: p = 0.005, quartic effect: p = 0.01) and much better than using the distance class (BIC = 300; main effect: p = 0.001.

For species-specific bat activity and despite the fact that either the model with ambient noise or with sky brightness seemed to predict activity quite well (Figs. S3, S4), these two models had model weights of close to 0 (BIC = 5169.0 and BIC = 5412.01, respectively) compared to the model with distance class (BIC = 4720.90; see Table S3 for the p-values of these models).

## Discussion

### Changes in bat communities along urban–rural gradients

According to our initial assumption, green areas most distant from the urban core were associated with greater species richness, species diversity as well as higher commuting, and foraging activity of bats. High levels of urbanization (distance class I and II) were associated with decreased bat activity in most observed species (apart from *Pipistrellus pipistrellus*, *Eptesicus serotinus, Vespertilio murinus* and sonotype Nycmi, which were positively associated with urban core sites). Using the example of Berlin–Brandenburg, Starik and Göttert showed that urban and rural areas can be characterized by their own typical community pattern of bats^5^. In this present study, we support these findings and show that the number of species and species diversity are lower at the urban core compared to the rural areas. In general, such changes in bat species richness and/or diversity along urbanization gradients have already been reported in similar studies using acoustic monitoring. For example, Jung and Kalko described a species decline of 25 to 16 species from a neotropical forest interior over a small town into urban habitats^10^. Similarly, Avila-Flores and Fenton compared bat activity between natural forested areas and urban settings and also found evidence for decreasing species richness in residential areas of Mexico City^12^. This monotonous relationship is further confirmed by studies conducted in Australia^38,39,60^ and also temperate environments^13,61^. However, there are also studies where this relationship could not be clearly established^65,74,75^. One explanation could be that the simple gradient is not always an appropriate tool to capture the changes occurring in bat communities along an urban–rural gradient. Alternatively, it could be that the responses of bat communities towards urban–rural habitat changes are case-specific so that there is not a common response pattern. In our current study we also aimed to describe the documented overall change in the bat community using a simple geographic linear urban-to-rural gradient which could potentially serve as a blueprint for other studies and possibly even for other urban settings.

### Distance classes as a meaningful methodological design to assess the effects of urbanization on biodiversity in the region of Berlin–Brandenburg

Our experimental approach seems relatively simple—from the geographical city center we defined urbanization categories based on distance (5 km intervals) along a linear transect westwards to the outskirts of the urban matrix and into the rural surrounding of the metropole. However, although this approach seems useful and simple to implement, it has not been used before, perhaps because different administrative units leading to a higher effort when applying for the relevant permits, as each district has its specific requirements. The study sites associated with distance classes I–III lie within the geographical-administrative boundaries of the city of Berlin, while the study sites of distance classes IV to VI are already located in the federal state of Brandenburg. The statistical exploration of candidate predictor variables (PCAs) supports this methodological approach showing that many variables of urbanization correlated with the urbanization gradient. Our main results illustrate that the changes in the bat community can be assessed very well with our established hypothetical Berlin-specific gradient: the distance classes are a meaningful predictor variable for the degree of urbanization in our specific example, as they already illustrate all measured variables of urbanization. In an attempt to investigate the respective importance of the effects of light, noise, and other characteristics that may be reflected by the distance class, we calculated three additional different models. Although an effect of light and noise is detectable for all species, the model using distance classes described our data more accurately compared to the models using light and noise as alternative predictors (as seen in the BIC values) except for species diversity where ambient noise was the best predictor.

With regard to our study, the observed decrease in diversity and species richness from the urban core to the rural areas can obviously be attributed to the specific geo-spatial and geo-historical situation and the settlement structure of the city itself. Berlin provides a unique case study as it resembles a metropolis that fits into a sparsely populated rural matrix and thus corresponds to a textbook urbanization gradient rarely encountered in other metropolitan areas^36^. Thus, the situation in Berlin might be completely different from e.g., South American São Paulo, Australian Sydney, North American New York or European Rome and we are aware that our design with these specific distance classes does not necessarily apply to metropolitan areas elsewhere. However, our study encourages further research on urban–rural gradients based on such simple methodological approaches, for example in urban areas with a similar geospatial structure. We believe that the example of Berlin–Brandenburg is particularly suitable for investigating the impact of urbanization on biodiversity, also with regard to other organismic groups for which gradient-dependent response patterns have been reported, for example some representatives of the order Rodentia^76^.

### Sudden shift in communities occurring within the urban matrix—suburban habitats as “buffer zones” for bats?

Contrary to our assumption of a gradual turnover of bat community characteristics along a linear gradient, we found significant changes and sudden shifts of parameters occurring on a relatively limited part of the overall gradient and at a point, where we did not expect it (at the transition between urban core and suburban matrix). On the one hand, this supports the chosen gradient from Berlin urban core to very rural Brandenburg to span sufficient space to adequately capture existing urbanization effects on bats. On the other hand, the results strongly suggest, that although the exemplarily measured “negative” structural effects of urbanization recede at a relatively late stage (i.e., light and noise pollution somewhat in distance class IV and more clearly in distance class V and VI), effects on the bat community become visible closer to the center (in distance class III). According to the ecotone hypothesis^77,78^, the border between two adjacent habitats, such as city and rural surroundings, can be characterized by very strong edge effects. Thus, one could also assume that such edge effects can occur at the border between Brandenburgs’ countryside and Berlins’ city areas, which then would be reflected by a very species-rich and diverse transition area (the ecotone, which would correspond to transition between distance class III and IV in our study). However, our observations indicate that the community changes already occur between distance class II and III (i.e. already within the outer city districts in the urban context at the transition between urban and peri-urban areas), and only to a lesser extent at the actual urban–rural border (transition from peri-urban to rural). Therefore, if bats are to be used as bioindicators for the effects of urbanization on biodiversity in the future, one should be aware that bats obviously do not exhibit a gradual adaptation corresponding to the degree of anthropogenic disturbance variables. More likely, effects on this species group will be “buffered” by the peri-urban areas. According to this, the suburban and peri-urban green areas of Berlin appear to serve as habitat refugia for specific bat species like *Myotis nattereri*, *Nyctalus leisleri* or *Plecotus* sp., which are characterized by visible increase in both commuting and foraging activity from distance class IV towards distance class VI. We assume that, contrary to the ecotone in natural habitats, in urban habitats the “edge zone or boundary line effect” arises primarily from anthropogenic influence due to the cities’ average small size. Following this, the “intermediate” environment between urban and rural areas (i.e. the ecotone) can become very large and its influence as a “buffer zone” can be significant.

### Winners vs losers of urbanization

Prior research on the effects of urbanization on bats has led to mixed results. In general, the majority of studies report a simplification/homogenization of bat communities^19^: while sensitive species are “filtered out”, species with more generalist traits prevail. As the effects of urbanization are different depending on species and context (as seen in Fig. 3, Table S3), our findings underline the necessity of species-specific considerations when evaluating effects of urbanization on the bat communities. It can be suspected that certain bat species respond more sensitively to urban-associated environmental changes than others conferring to their differential ecological and life history traits^19,40^. Accordingly, in our results this gradation of “sensitivity” is reflected by visible shifts in the community composition along the urbanization gradient and it seems that three groups can be clearly differentiated: we can support the existence of few “urban winners” (“urban exploiters” sensu McKinney 2006)^6^, such as the common pipistrelle *Pipistrellus pipistrellus* and the serotine bat *Eptesicus serotinus*, which actually increase in abundance within the urban core in a densely populated context. The more sensitive species, such as the noctule bat *Nyctalus noctula*, the soprano pipistrelle *Pipistrellus pygmaeus*, Nathusius’ pipistrelle *Pipistrellus nathusii* or Daubenton’s bat *Myotis daubentonii*, are able to exist within the urban boundaries (“urban adapters”) up to specific threshold levels for certain disturbance factors depending on the cities’ attributes. And finally, there are the “urban loser” species, such as Natterer’s bat *Myotis nattereri*, Leisler’s bat *Nyctalus leisleri* and species entailed within the sonotypes Plecotus (Brown long eared bat *Plecotus auritus* and Grey long-eared bat *Plecotus austriacus*) and Mkm (Brandt’s bat *Myotis brandtii* and whiskered bat *Myotis mystacinus*), that do not occur in the city center at all (“urban avoiders” sensu McKinney 2006)^6^.

*P*. *pipistrellus* and *E*. *serotinus* are known as “synanthropic species”^79,80^, dependent upon buildings with niches, crevices or various attics and their continuous occurrence in the densely built-up city center should not surprise. However, while the common pipistrelle actually seems to be resident in almost all larger cities in Germany, evidence of the serotine bat is found very frequently in settlements, but less frequently in the midst of a metropolitan matrix^81,82^. Thus, the occurrence and high activity levels of the serotine bat in the metropolitan center could also be specific to only a subset of European urban areas, where the rural matrix is located in relatively close spatial distance to the city. For *E*. *serotinus*, a certain proximity to (food) resources seems mandatory because this species faces difficulties in food acquisition in urban ecosystems that do not provide the same insect concentrations (qualitatively and quantitatively) as rural habitats. Also, Kervyn and Libois found that *E. serotinus* roosts in urban buildings but forages outside the city^83^. At this point, our results cannot inform the discussion whether *E. serotinus* and *P. pipistrellus* are actually better adapted to urban conditions or if they might benefit from the disappearance of other species according to the classic resource and niche concept^5,84^. Likewise, in regard to species with no visually apparent effect of urbanization (e.g., *Vespertilio murinus*), it remains questionable if this lack of effect is due to their overall low occurrence rather than an absence of impact. However, these considerations lead to the following consequence: when trying to analyze relationships between ecological factors or traits (e.g. life history, niche breadth, etc.) and urban success, there is an ultimate need to control for source population size in order to avoid the “wrong” correlations. For example, certain life history and ecological factors may have caused species to be rare in non-urban habitats of a given region, but may have no effect per se in avoiding urban areas. Likewise, the possibility that species may frequently inhabit urban areas simply because they occur more frequently in the city’s surrounding area cannot be ruled out.

Finally, “bottom-up regulations”, i.e. differential effects of urbanization on the different species’ prey availability and/or distribution as well as the amount of available habitat due to urban intensification might play a role that should not be neglected. For instance, results of Lewanzik and Voigt revealed that insect diversity increased both bat relative activity and diversity, which emphasizes such trophic control mechanisms^85^.

### Variability of bat species activity against the background of the different predictor variables

Similar to all reductionist concepts, the idea of the gradient is based on a certain simplification in order to grasp a biological effect with reasonable effort—given the complexity of biological phenomena, there is a risk that a conceptual simplification could distort or even obscure this effect. To better understand this, results from the reduced and full datasets were compared. For the reduced data set, the distance class models are most suitable to describe the changes in the number of species. Distance classes are therefore the best predictor of the occurrence of species. However, when looking at the diversity parameter of the community (Shannon index), the distance class model reaches its limits—here the models for light and noise are more suitable. Accordingly, there is a difference in the suitability of different models (distance classes vs. light and noise pollution), depending on whether quantitative (number of species) or qualitative (Shannon index) aspects are the focus. There is no one suitable model for the reduced data set, but the suitability of the models and thus the influence of various parameters depends on the question/level to be examined. It appears that autecological factors (light, noise) determine the composition of the community and the frequency of individuals per species most strongly, while the occurrence depends primarily on the more multifactorial features of the microhabitat (distance class).

The negative effect of artificially illuminated night sky on bat activity has been already reported^24,28,74,86,87^. Artificial lighting can modify the foraging behavior of bat communities^19^: while a subset of light-opportunistic species (e.g. *Pipistrellus pipistrellus*) can take advantage of insect resources at the expense of light-averse ones. With regard to noise pollution, it is known that bat activity is decreasing as background dB levels increase^88^ and that successful prey localization declines^3,89,90^. Thus, anthropogenic noise and artificial light at night may indeed determine the local distribution of bat species along an urban-rural gradient. Although in our study, the effects of ambient noise and artificial light were not as strong as the effect of the distance class according to the BIC values, the variation in bat activity seems still related to the two variables. Thus, ambient noise and artificial light seem to have a strong potential to predict bat species activity, but neither one nor the other alone seems to sufficiently explain our data as the distance class still provides the much better prediction here (Table Supplement S3). As the “noise model” seems slightly better than the “light model” according to the BICs, we assume that sensory variation (e.g. different hearing sensitivity) among bat species may determine the different sensitivity towards noise disturbance levels^15^. For example, bat species that hunt by listening to the sounds of their prey should be much more sensitive to low frequency anthropogenic sounds than bats that only use their own echolocation for hunting^91^. Unfortunately, we cannot conclusively clarify the discussion about the causes of the species distribution and the influences of the disturbance variables at this point. With regard to future studies, we therefore suggest to investigate the influence of these two variables on the basis of hourly values for all measured variables in order to disentangle the effects of ambient noise, artificial light and the distance class.

The full data set shows a clearer pattern than the reduced data set, suggesting the importance of an adequate study period to capture the influence of urbanization intensity on bat communities. However, the question of the extent to which the effect can be attributed to the distance classes or the noise and light pollution cannot be answered with the entire data set because noise and light data are not available for all nights.

## Conclusion

Using a spatially linear gradient and a comprehensive data set of two years, our study provided an interesting perspective on the vulnerability, sensitivity and adaptability of European bat species towards urbanization intensity. For the specific example of Berlin–Brandenburg, our chosen urban–rural gradient based on distance classes enabled us to capture the effects of urbanization on bat communities. We observed a sudden shift in the bat community occurring closer to the city center than we thought, i.e., closer to the city center than the transition between urban and peri-urban areas. This suggests a significant influence of this intermediate environment as a “buffer zone” for specific bat species. Our results imply that most species are not able to adapt to anthropologically modified landscapes, specifically to the inner core of the metropolitan area, possibly due to high levels of specific disturbance factors. Although we could demonstrate that anthropogenic noise and artificial light have the potential to predict the variability of bat species activity along the urban–rural gradient, the actual influence on observed shifts in the bat community needs further research.

### Supplementary Information

Supplementary Information.

## Data Availability

We archived all data on an open-source cloud-based project management platform (Open Science Framework, OSF) (https://osf.io/bt4rf/?view_only=feaa2e6d20814bdab1102e7f00f1bda7). These data were all archived under a Creative Commons license (CC-BY-NC), making them open-access to the community. All the data supporting this work are available from the corresponding author upon reasonable request.
