# Supplementary material for: Unexpected bat community changes along an urban–rural gradient in the Berlin–Brandenburg metropolitan area
Source: Sci Rep. 2024 May 8;14:10552. doi: 10.1038/s41598-024-61317-7 (PMC11078944; doi:10.1038/s41598-024-61317-7)
Supplement: Supplementary file 1 — Supplementary Information. [file 41598_2024_61317_MOESM1_ESM.docx]

**Supplementary Information for**

**Unexpected bat community changes along an urban-rural gradient in the Berlin-Brandenburg metropolitan area**

Nicole Starik ^1,2^, Lorenz Gygax^1^ and Thomas Göttert ^3^*

^1^Albrecht Daniel Thaer-Institute of Agricultural and Horticultural Sciences, Faculty of Life Sciences, Humboldt-Universität zu Berlin, 10009 Berlin, Germany; nicole.starik@hu-berlin.de

^2^Deutsche Fledermauswarte e.V., Am Juliusturm 63, 13599 Berlin, Germany.

^3^ Research Center [Sustainability–Transformation–Transfer], Eberswalde University for Sustainable Development, 16225 Eberswalde, Germany; thomas.goettert@hnee.de

*Correspondence: thomas.goettert@hnee.de; Tel.: +49 3334 657 521

**Data availability**

We archived all data on an open-source cloud-based project management platform (Open Science Framework, OSF; https://osf.io/bt4rf/?view_only=feaa2e6d20814bdab1102e7f00f1bda7). These data were all archived under a Creative Commons license (CC-BY-NC), making them open-access to the community.

**Supporting methods**

Table S1. Measured parameters at study sites (n= 18 green area sites in Berlin and Brandenburg district). Site characteristics were measured once during study, night characteristics were measured for a selection of nights (n=5) per site. The variables % impervious surface, mean height of buildings in 100m radius, ambient noise, and sky brightness were chosen as they characterize the level of urbanization and/or disturbance; the variables distance to water, distance to nearest tree, distance to nearest building, and mean height of vegetation were chosen as they reflect environmental conditions, which are likely to affect the access to important resources for bats.

| **distance class** | | | **I** | | | **II** | | | **III** | | | **IV** | | | **V** | | | **VI** | | |
| --- | --- | --- | --- | --- | --- | --- | --- | --- | --- | --- | --- | --- | --- | --- | --- | --- | --- | --- | --- | --- |
| **study site** | | | **1** | **2** | **3** | **4** | **5** | **6** | **7** | **8** | **9** | **10** | **11** | **12** | **13** | **14** | **15** | **16** | **17** | **18** |
| **site-specific**  **characteristics** | distance to (geographical) city centre (km) | | 2.4 | 2.2 | 3.3 | 9.2 | 7.8 | 6.9 | 11.1 | 16.5 | 14.3 | 19.1 | 19.6 | 18.8 | 25.2 | 24.9 | 27.2 | 32.6 | 41.9 | 33.2 |
|  | % impervious surface in 500m radius | | 72.31 | 68.14 | 85.74 | 69.8 | 71.5 | 66.5 | 60.8 | 54.5 | 53.2 | 43.5 | 49.5 | 56.5 | 49.9 | 43.4 | 47.8 | 42.98 | 50.13 | 47.82 |
|  | mean height of buildings in 100m radius (m) | | 16.2 | 19.8 | 21.3 | 22.0 | 19.3 | 18.9 | 17.8 | 16.2 | 15.9 | 7.3 | 9.4 | 12.5 | 8.2 | 7.5 | 6.9 | 5.8 | 8.6 | 7.6 |
|  | distance to nearest building (m) | | 82 | 59 | 49 | 180 | 62 | 86 | 43 | 149 | 63 | 23 | 35 | 46 | 51 | 53 | 37 | 50 | 43 | 63 |
|  | distance to water (m) | | 89 | 101 | 33 | 253 | 69 | 32 | 161 | 190 | 59 | 213 | 62 | 237 | 69 | 102 | 270 | 423 | 493 | 529 |
|  | distance to nearest tree (m) | | 68 | 45 | 52 | 27 | 36 | 21 | 27 | 43 | 25 | 33 | 29 | 72 | 26 | 49 | 32 | 78 | 37 | 56 |
|  | mean height of vegetation in 100m radius (m) | | 14.5 | 16.6 | 15.1 | 13.7 | 16.6 | 15.8 | 17.5 | 11.4 | 14.5 | 17.5 | 14.3 | 13.4 | 17.9 | 18.9 | 15.5 | 21.4 | 18.5 | 16.8 |
| **night-specific characteristics** | ambient noise  (dB re20µPa) | median | 56.0 | 47.2 | 57.8 | 50.92 | 50.1 | 51.49 | 47.9 | 48.8 | 50.3 | 39.3 | 39.9 | 39.5 | 26.4 | 28.96 | 29.2 | 24.1 | 26.02 | 29.6 |
|  |  | range | 10.1 | 7.9 | 8.2 | 6.3 | 3.5 | 5.2 | 4.8 | 2.3 | 4.3 | 2.9 | 6.6 | 1.9 | 3.2 | 1.5 | 2.0 | 1.8 | 2.1 | 1.7 |
|  | sky brightness  (mag arcsec^2^) | median | 16.06 | 16.0 | 15.4 | 16.31 | 15.4 | 15.45 | 16.7 | 16.6 | 16.4 | 16.9 | 17.2 | 17.1 | 18.9 | 18.4 | 18.6 | 18.9 | 18.9 | 18.08 |
|  |  | range | 1.6 | 1.4 | 1.02 | 1.5 | 0.9 | 1.3 | 0.45 | 0.9 | 1.9 | 2.8 | 3.2 | 2.6 | 1.3 | 0.9 | 0.6 | 0.4 | 1.2 | 0.8 |

**Supporting results**

Table S2. Factor loadings from the Principal Component Analyses (PCA) performed with variables used to describe site and night specific characteristics of study sites

| **Dataset 1**  **(“site specific characteristics”, measured once for**  **each site)** |  |  |
| --- | --- | --- |
| **Importance of components** | **Comp.1** | **Comp.2** |
| Proportion of Variance | 0.5359682 | 0.1700262 |
| Cumulative Proportion | 0.5359682 | 0.7059944 |
| **Loadings** |  |  |
| distance class  [DistClass] | **0.493** | 0.039 |
| impervious surface in a 500 m radius (%)  [ImpervSurface] | **-0.452** | 0.096 |
| minimum distance of site to next building (m)  [DistBuild] | -0.256 | **0.556** |
| mean height of buildings in 100m radius (m)  [HeightBuild] | **-0.488** | 0.079 |
| minimum distance of site to next water body (m)  [DistWater] | 0.353 | **0.526** |
| minimum distance of site to next tree (m)  [DistTree] | 0.131 | **0.603** |
| mean height of vegetation in 100m radius (m)  [HeightVegetation] | 0.327 | 0.079 |
|  |  |  |
| **Dataset 2**  **(“night specific characteristics”,** **measured during**  **5 nights per site)** |  |  |
| **Importance of components** | **Comp.1** |  |
| Proportion of Variance | 0.9317674 |  |
| Cumulative Proportion | 0.9317674 |  |
| **Loadings** |  |  |
| distance class  [DistClass] | **0.582** |  |
| mean level of ambient noise per night (dB re20µPa)  [noise] | **-0.579** |  |
| mean level of sky brightness per night (mag arcsec^2^)  [light] | **0.571** |  |


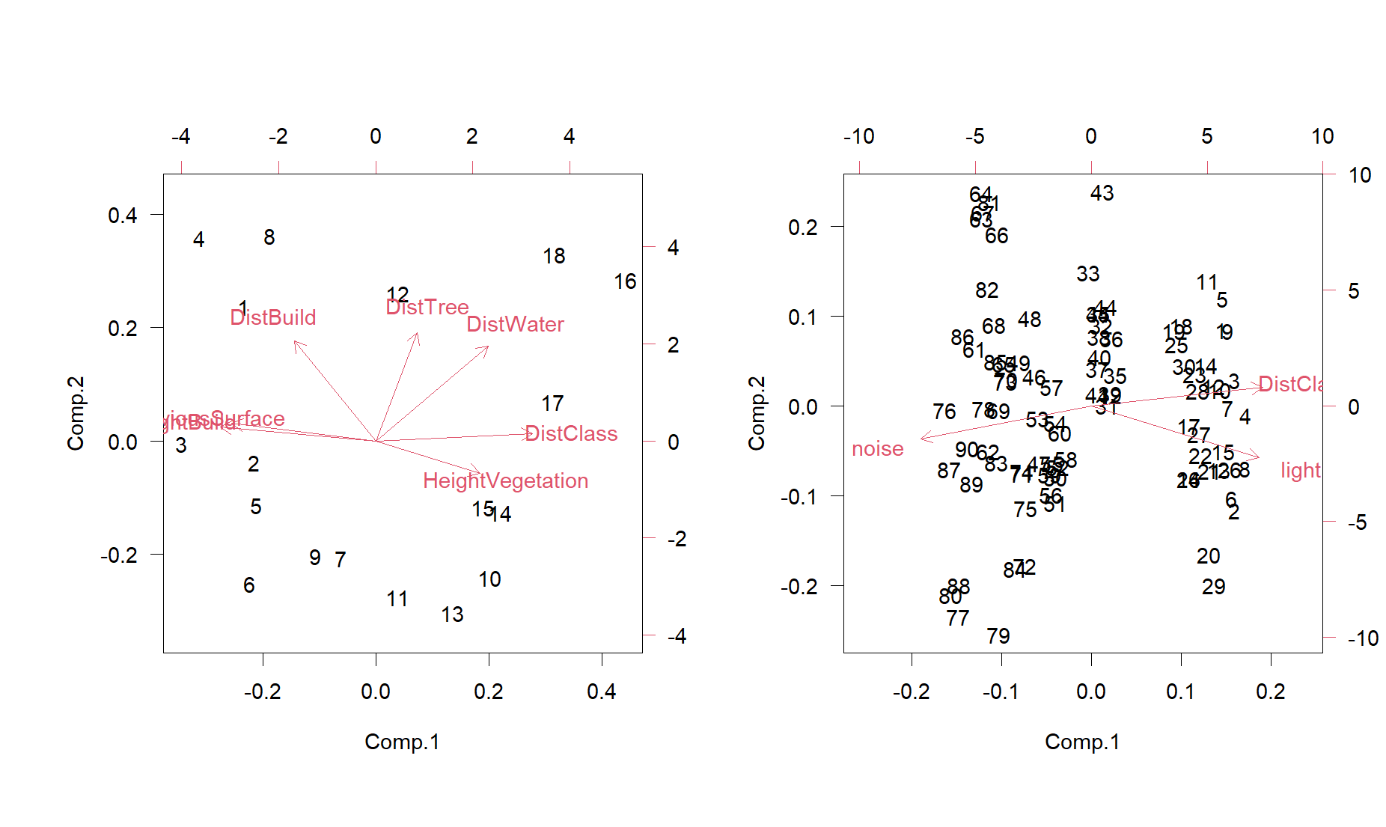


Figure S1. Principal component analysis (PCA) biplots of study sites based on two different data sets according to above Table S2: left) site-specific components and right) night-specific components of the urbanization gradient. Each data set was complemented by a continuous variable reflecting the urbanization gradient with numbers from 1 (urban core: ≤ 5km distance to geographical city center) to 6 (most rural: ≥30km distance to geographical city center).


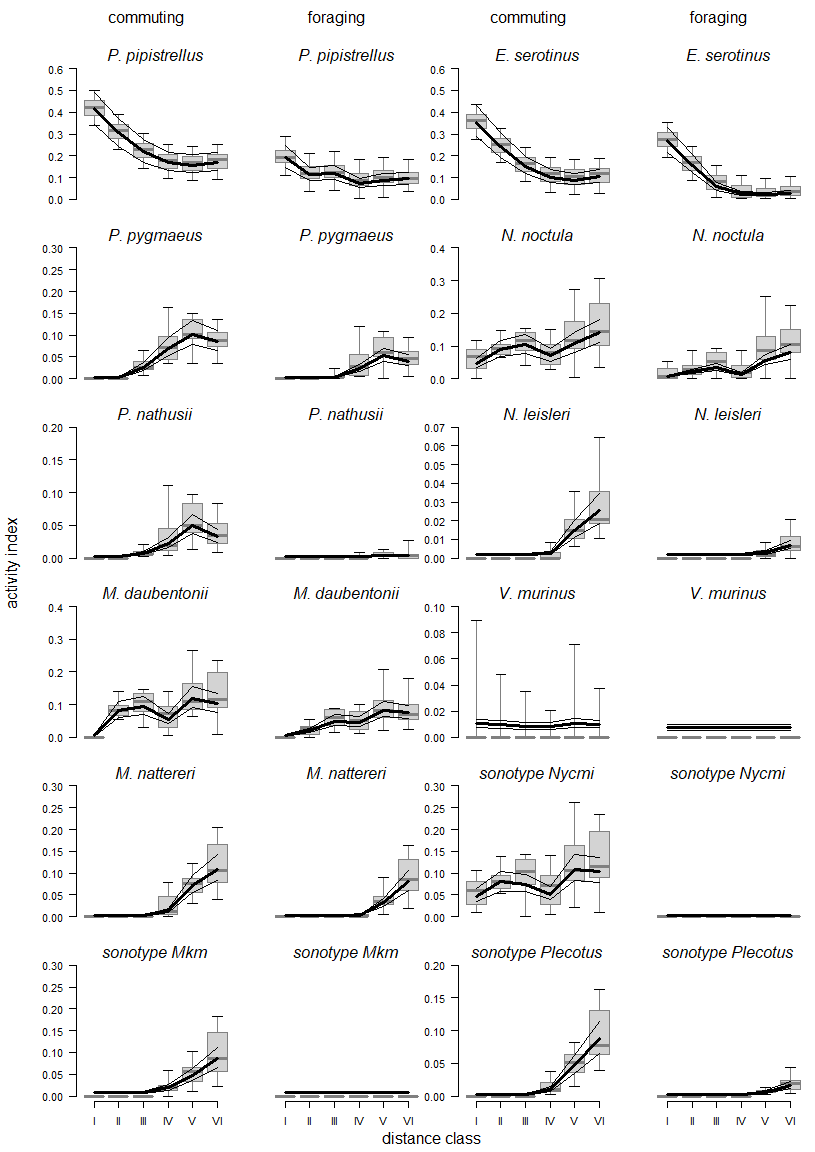


Figure S2. Species-specific commuting and foraging activity of bats in relation to the distance classes reflecting the urban-rural gradient in the same data set as used for Figures S3 and S4. Boxplots represent medians, quartiles and the total ranges. Thick lines: Model estimate; thin lines: 95% confidence intervals. Please note the different scales of the Y-axes.


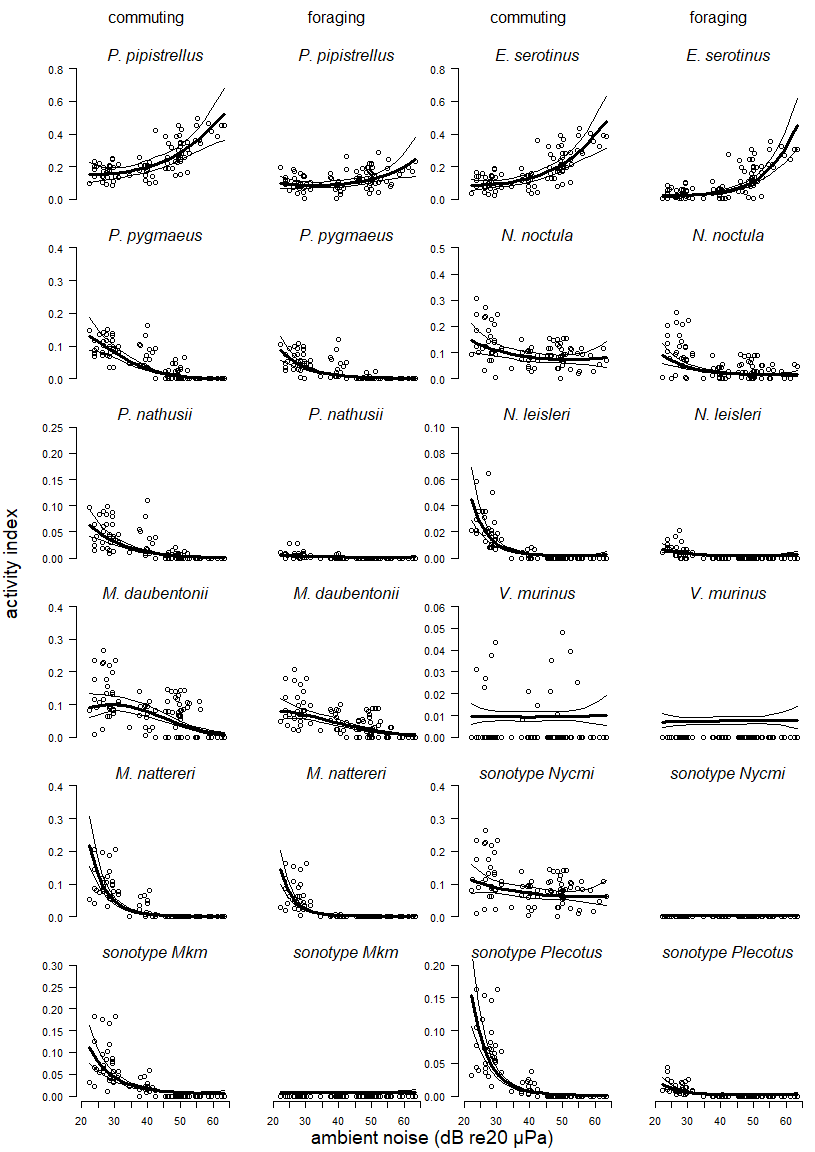


Figure S3. Species-specific commuting and foraging activity of bats in relation to ambient noise. Scatterplot of bat activity levels and night-specific measurements of noise levels. Thick lines: Model estimate; thin lines: 95% confidence intervals. Please note the different scales of the Y-axes.

*
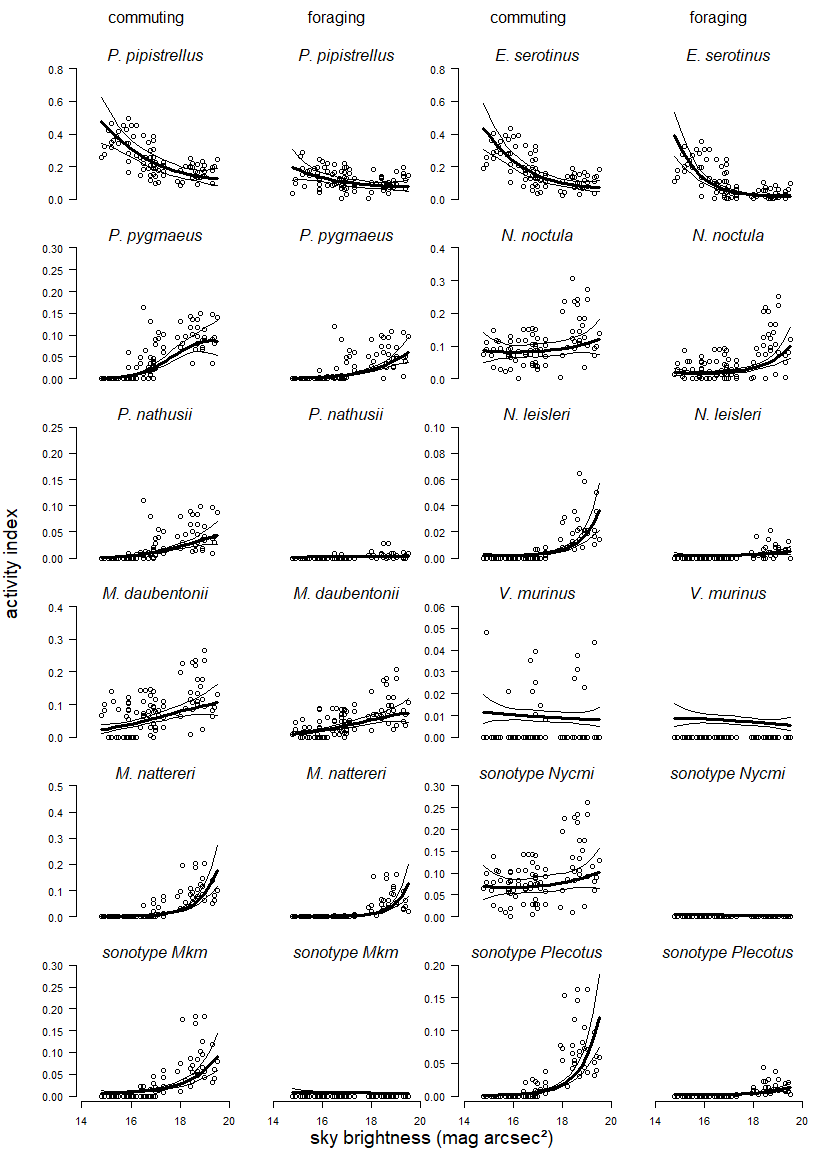
*

*Figure S4.* *Species-specific commuting and foraging activity of bats in relation to sky brightness. Scatterplot of bat activity levels and night-specific measurements of sky brightness as a proxy for artificial light pollution; values >20 mag/arcsec^2^ would indicate a completely clear sky with no light pollution, as the number gets lower, so does the visibility of stars. Thick lines: Model estimate; thin lines: 95% confidence intervals. Please note the different scales of the Y-axes.*

*Table S3*

*Global p-values as well as p-values of main effects and interactions; p-values based on parametric bootstrapping.*

|  |  | **data set 1**^a^ | **data set 2**^b^ | | |
| --- | --- | --- | --- | --- | --- |
| **Main fixed effect** (mfe) | | **distance class** (categorical) | **distance class** (categorical) | **ambient noise** (continuous) | **sky brightness** (continuous) |
| global test |  | 0.001 | 0.001 | 0.001 | 0.001 |
| mfe |  | 0.001 | 0.001 | 0.001 | 0.002 |
|  | *context | 0.001 | 0.001 | 0.001 | 0.001 |
|  | *species | 0.001 | 0.001 | 0.001 | 0.001 |
|  | *species*context | 0.001 | 0.001 | 0.001 | 0.001 |
| mfe (squared) | | – | – | 0.002 | 0.06 |
|  | *context | – | – | 0.13 | 0.14 |
|  | *species | – | – | 0.001 | 0.001 |
|  | *species*context | – | – | 0.02 | 0.02 |
| species |  | 0.001 | 0.001 | 0.001 | 0.001 |
|  | *context | 0.001 | 0.001 | 0.001 | 0.001 |
| context |  | 0.003 | 0.002 | 0.001 | 0.001 |

*^a^ full data set
^b^ reduced data set (only including those nights with noise / light measurements)*
